# Identification of an R2R3-MYB gene regulating tepal background coloration in *Tricyrtis* sp.

**DOI:** 10.1038/s41598-026-46254-x

**Published:** 2026-03-28

**Authors:** Yuta Shinoku, Ichiro Kazama, Yusuke Kanemaki, Mai Shibuya, Kakeru Inagawa, Julia Ono, Masaru Nakano, Masahiro Otani

**Affiliations:** 1 Graduate School of Science and Technology, Niigata University, 2-8050 Ikarashi, Nishi-ku, Niigata, 950-2181 Japan; 2 Faculty of Agriculture, Niigata University, 2-8050 Ikarashi, Nishi-ku, Niigata, 950-2181 Japan

**Keywords:** Anthocyanin, Liliaceous ornamental plant, RNAi, Flower color pattern, Shading, Biotechnology, Molecular biology, Plant sciences

## Abstract

The liliaceous ornamental plant *Tricyrtis* sp. produces unique flowers, whose tepals have many random reddish-purple spots on a light purple background. In our previous studies, we performed comprehensive isolation and expression analysis of the anthocyanin biosynthesis-related genes to elucidate the molecular mechanism underlying flower color pattern formation in this plant, and identified the R2R3-MYB transcription factor gene, *TrMYB1*, expressed in tepals. In the present study, we carried out detailed expression and functional analyses of *TrMYB1*. Shading treatment of flower buds markedly reduced background coloration of the tepals, while there was little effect on spot formation. In addition, *TrMYB1* expression level decreased in the tepals of shaded flower buds. Overexpression of *TrMYB1* in *Tricyrtis* sp. resulted in deeper coloration and significantly increased anthocyanin content in tepals. RNAi-mediated knockdown of *TrMYB1* significantly suppressed the expression of the anthocyanin biosynthetic enzyme genes, resulting in a significant decrease in anthocyanin contents and marked reduction in background coloration in the tepals, with little effect on spot formation. These results indicate that tepal background coloration and spot formation may be regulated by different molecular mechanisms, and that background coloration is likely induced by light through activation of *TrMYB1*.

**Supplementary Information:**

The online version contains supplementary material available at 10.1038/s41598-026-46254-x.

## Introduction

Flower color and flower color pattern are among the most important traits for ornamental plants. Flower color is determined primarily by the type and concentration of pigments, as well as the combination of multiple pigments. Whereas flower color patterns, such as spots, variegation, stripes, picotee, and gradation, are caused by different patterns of pigment accumulation in tepals^1^. Among flower pigments, anthocyanins, members of flavonoids, are found in various plant species and are responsible for a wide range of colors including orange, red, purple, and blue^2,3^.

The anthocyanin biosynthetic pathway is broadly conserved among higher plant species and is well understood^3^. In this pathway, eight enzymes, chalcone synthase (CHS), chalcone isomerase (CHI), flavanone-3-hydroxylase (F3H), flavonoid 3′-hydroxylase (F3′H), flavonoid 3′,5′-hydroxylase (F3′5′H), flavonol synthase (FLS), dihydroflavonol 4-reductase (DFR), and anthocyanin synthase (ANS) are involved in the biosynthesis of anthocyanidins and flavonols^3,4^. The expression of these enzyme genes is known to be mainly regulated by the MBW transcription factor complex consisting of R2R3-myeloblastosis (MYB), basic helix-loop-helix (bHLH), and WD40 repeats (WDR) proteins^5^. These transcription factors and their functions have also been characterized in various higher plant species^3,5,6^.

Anthocyanin biosynthesis is known to be affected by environmental stimuli such as light, temperature, and drought. Among them, light has been extensively studied as a key factor influencing anthocyanin biosynthesis^7^. It has been reported that shading treatments reduce anthocyanin accumulation in tepals or fruit skins in *Eustoma grandiflorum*^8^, *Gerbera hybrida*^9^, *Malus domestica*^10^, *Chrysanthemum morifolium*^11^, *Solanum melongena*^12^, and *Lilium regale*^13^. The molecular mechanisms underlying light-induced anthocyanin biosynthesis have been investigated in several plant species. In *Petunia hybrida*, both an R2R3-MYB transcription factor *PHZ* and a bHLH transcription factor *AN1* are expressed in response to light and play key roles in regulating anthocyanin biosynthesis in floral and vegetative organs^14,15^. In *L*. *regale*, light-induced R2R3-MYB factor *LhMYB15* is implicated in the regulation of anthocyanin biosynthesis, contributing to characteristic pigmentation pattern formation in tepals^13^. In *C*. *morifolium*, light induces the expression of *CmMYB9a* while suppressing that of *CmBBX28*, a B-box protein that negatively regulates anthocyanin biosynthesis, thereby leading to anthocyanin accumulation in petals^16^.

*Tricyrtis* spp. are liliaceous ornamental plants native to Japan. One of the *Tricyrtis* cultivars, ‘Shinonome’, produces unique flowers with tepals bearing numerous randomly distributed reddish-purple spots on a light purple background. This flower color pattern is clearly different from variegation patterns caused by transposons^17–19^ and bicolor patterns caused by post-transcriptional gene silencing^20,21^. To elucidate the molecular mechanism of flower color pattern formation in *Tricyrtis* sp. ‘Shinonome’, we performed comprehensive isolation and expression analysis of the anthocyanin biosynthesis-related genes in this plant^1,22^. To date, seven flavonoid biosynthetic enzyme genes, *TrCHS* (AB478624; GenBank/EMBL/DDBJ databases; the same applies below), *TrCHI* (AB908277), *TrF3H* (LC209222), *TrF3*′*H* (AB480691), *TrDFR* (AB830112), *TrFLS* (LC103181), and *TrANS* (LC209106), and three transcription factor genes for the MBW complex, *TrMYB1* (AB856412), *TrbHLH2* (LC223741), and *TrWDR* (LC223742), have successfully been isolated. The expression of *TrMYB1* in tepals is correlated with that of ‘late’ biosynthetic enzyme genes (*TrF3’H*, *TrDFR*, *TrFLS* and *TrANS*), suggesting that *TrMYB1* may contribute to anthocyanin biosynthesis in tepals through regulation of these genes. However, the role of *TrMYB1* in flower color pattern formation remains unclear.

In the present study, we examined the effects of light on anthocyanin biosynthesis and the expression of *TrMYB1* in the tepals of *Tricyrtis* sp. ‘Shinonome’ by shading treatment of flower buds, and further analyzed the function of *TrMYB1* through overexpression and RNAi-mediated knockdown in transgenic plants.

## Result

### Shading treatment of flower buds in *Tricyrtis* sp. ‘Shinonome’

**Fig. 1:**
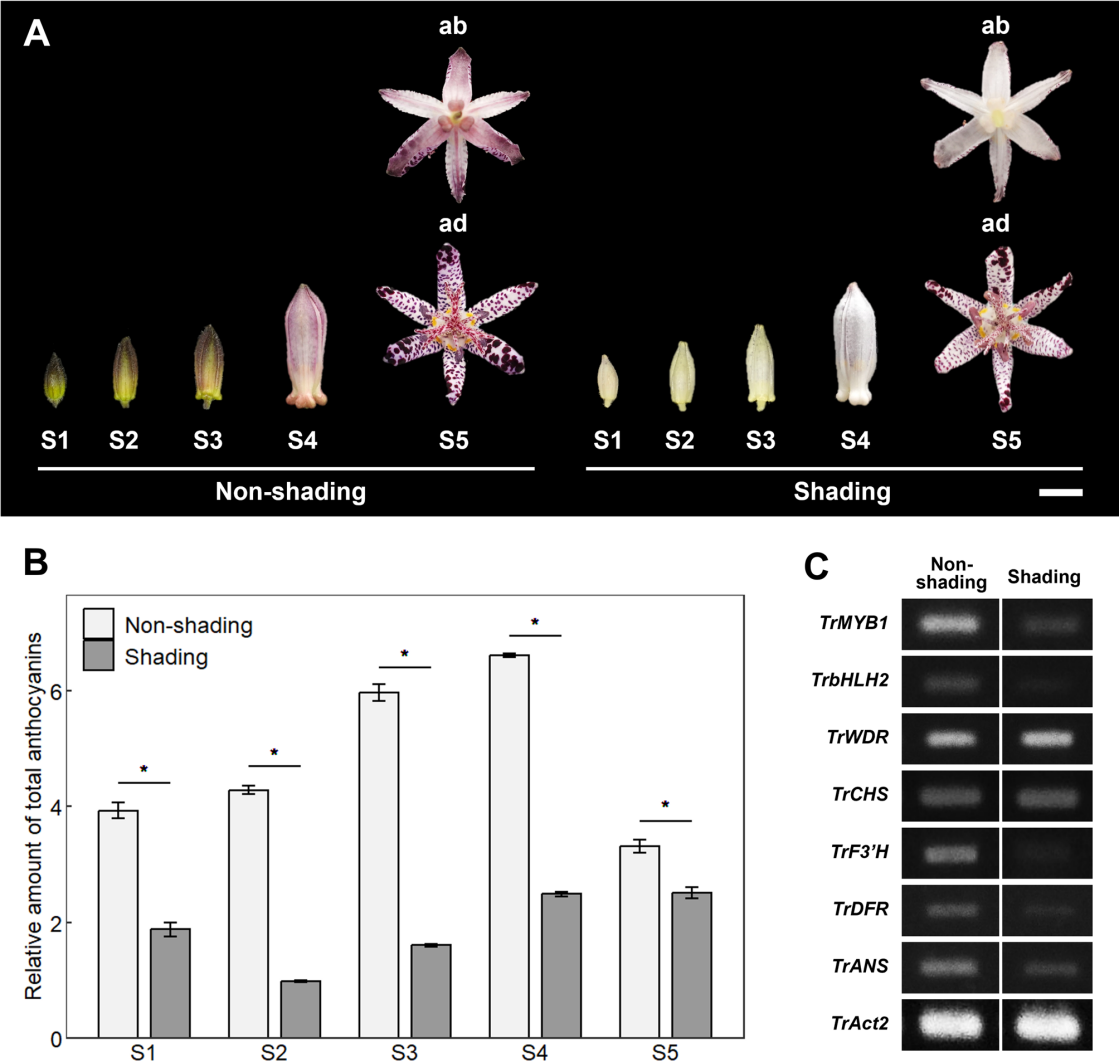
Effects of shading treatment on flower buds in *Tricyrtis* sp. (**A**) Representative flower phenotypes at each developmental stage under non-shaded and shaded conditions. ab, abaxial side; ad, adaxial side. Bar = 1 cm. (**B**) Relative amounts of total anthocyanins in outer tepals at each developmental stage under non-shaded and shaded conditions. Data are shown as means ± standard errors (SE; n = 9). * indicates significant differences at *p* < 0.001 by Student’s t-test. (**C**) Expression levels of anthocyanin biosynthesis-related genes in outer tepals at S4 under non-shaded and shaded conditions. *TrAct2* was used as an internal control. Cropped gel images are shown, and the corresponding full-length gel images are provided in Supplementary Figure S2.

To examine the effects of light on anthocyanin accumulation and the expression of anthocyanin biosynthesis-related genes in tepals of *Tricyrtis* sp. ‘Shinonome’, shading treatment of flower buds was carried out. Under non-shaded conditions, background coloration in tepals was observed from S1 flower buds onwards particularly on the abaxial side of the outer tepals (Fig. 1A). Spot formation was detectable in tepals of the S1 flower buds and expanded over the entire tepals from S2 onwards (Supplementary Fig. S1). In S5 flowers, tepals were characterized by numerous randomly distributed reddish-purple spots on a light purple background. On the other hand, under shaded conditions, background coloration in tepals was suppressed in S1–S4 flower buds (Fig. 1A). In S5 flowers, tepal background coloration was much reduced compared with those under non-shaded conditions, although reddish-purple spots were produced in the same manner as under non-shaded conditions (Fig. 1A).

Under shaded conditions, the relative amounts of total anthocyanins in outer tepals significantly decreased at all stages of flower development compared with those under non-shaded conditions (Fig. 1B). Real-time RT-PCR analysis for the expression of anthocyanin biosynthesis-related genes in outer tepals of S4 flower buds revealed that shading treatment reduced the expression levels of *TrMYB1*, *TrbHLH2*, *TrF3’H*, *TrDFR*, and *TrANS* (Fig. 1C). On the other hand, no apparent differences in the expression level of *TrWDR* and *TrCHS* were observed between shaded and non-shaded conditions (Fig. 1C). In inner tepals, background coloration was inherently faint, making the effect of shading less apparent than in outer tepals (Supplementary Fig. S2).

### Promoter isolation and cis-element analysis of *TrMYB1*

To investigate whether *TrMYB1* expression may be regulated by light, the upstream promoter region (− 2934 bp) of *TrMYB1* was isolated, and cis-elements in the promoter sequence were analyzed. Several putative light-responsive cis-elements, including G-box, LAMP-element, AE-BOX, Box 4, and GT1-motif, were identified (Supplementary Fig. S3).

### Overexpression of *TrMYB1* in *Tricyrtis* sp. ‘Shinonome’

**Fig. 2:**
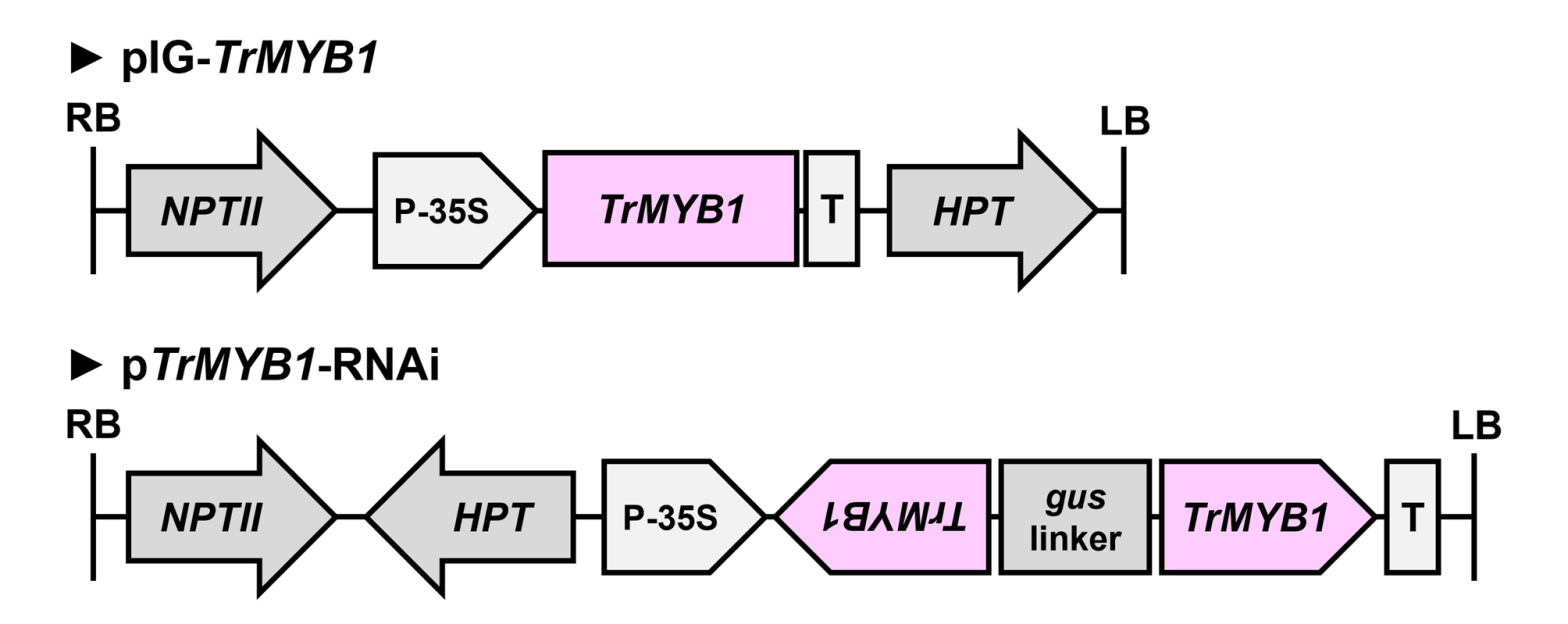
T-DNA regions of the vectors used for transformation. T-DNA regions of the overexpression vector (pIG-*TrMYB1*) and the RNAi-mediated knockdown vector (p*TrMYB1*-RNAi). *gus* linker, an internal fragment of the β-glucuronidase (GUS) gene connecting trigger sequences; *HPT*, hygromycin phosphotransferase gene driven by the CaMV35S promoter; LB, left border; *NPTII*, neomycin phosphotransferase II gene driven by the *NOS* promoter; P-35S, CaMV35S promoter; RB, right border; T, *NOS* terminator; *TrMYB1*, full-length cDNA or trigger sequence of *TrMYB1*.

**Fig. 3:**
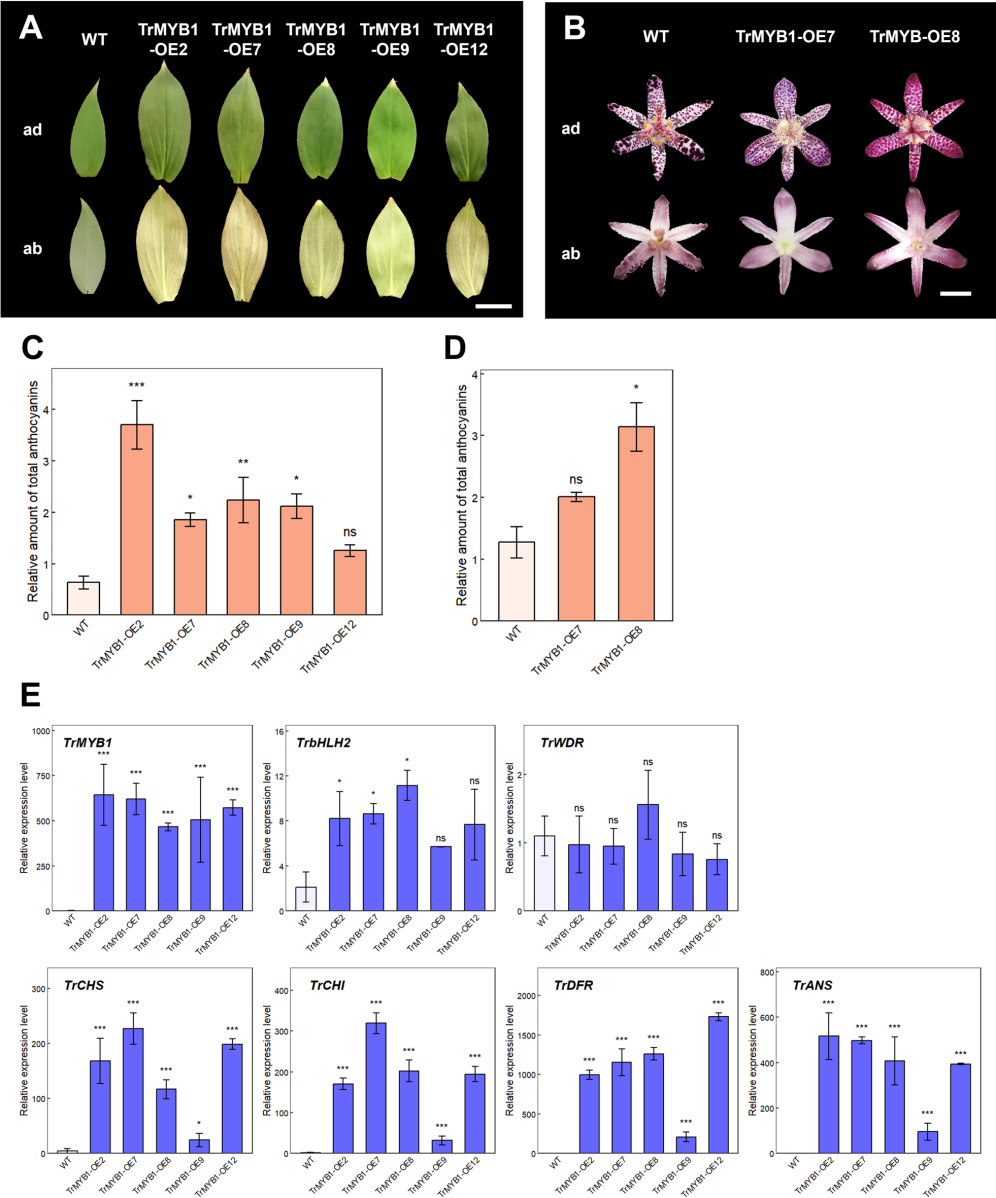
Effects of *TrMYB1* overexpression in transgenic plants (**A**) Representative leaf phenotypes of WT and transgenic plants. Bar = 2 cm. (**B**) Phenotypes of flowers of WT and transgenic plants. Bar = 1 cm. ab, abaxial side; ad, adaxial side. (**C**) Relative amounts of total anthocyanins in leaves of WT and transgenic plants. Data are shown as means ± SE (n = 3). *, ** and *** indicate significant differences compared with WT at *p* < 0.05, *p* < 0.01 and *p* < 0.001, respectively, by Dunnett’s test following one-way ANOVA. ns, not significant. (**D**) Relative amounts of total anthocyanins in outer tepals of WT and transgenic plants. Data are shown as means ± SE (n = 3). * indicates significant differences compared with WT at *p* < 0.01 by Dunnett’s test following one-way ANOVA. ns, not significant. (**E**) Relative expression levels of anthocyanin biosynthesis-related genes in leaves of WT and transgenic plants by real-time RT-PCR. Data are shown as means ± SE (n = 3). *, ** and *** indicate significant differences compared with WT at *p* < 0.05, *p* < 0.01 and *p* < 0.001, respectively, by Dunnett’s test following one-way ANOVA. ns, not significant.

To assess the effect of *TrMYB1* overexpression, *TrMYB1* under the control of the cauliflower mosaic virus (CaMV) 35S promoter was introduced into *Tricyrtis* sp. ‘Shinonome’ (Fig. 2). Five independent transgenic lines (TrMYB1-OE2, -OE7, -OE8, -OE9, and -OE12) were obtained (Fig. 3A), among which TrMYB1-OE7 and -OE8 produced flowers (Fig. 3B). These transgenic lines showed enhanced pigmentation in most floral organs as well as in leaves compared with the wild type (WT) (Fig. 3A, B). However, no obvious pigmentation was observed in the basal region of the tepals. In contrast, pigmentation was markedly enhanced in naturally pigmented regions of the tepals of wild-type plants (Fig. 3B). In particular, TrMYB1-OE2, -OE7, -OE8, and -OE9 showed significantly increased anthocyanin accumulation in leaves (Fig. 3C), and TrMYB1-OE8 showed significantly increased anthocyanin accumulation in outer tepals of S5 flowers (Fig. 3D). Real-time RT-PCR analysis using leaves showed that the expression levels of *TrMYB1* and enzyme genes (*TrCHS*, *TrCHI*, *TrDFR*, and *TrANS*) significantly increased in all five transgenic lines (Fig. 3E). *TrbHLH2* expression also significantly increased in TrMYB1-OE2, -OE7, and -OE8, whereas no significant changes in *TrWDR* expression levels were observed in any of the five transgenic lines (Fig. 3E).

### RNAi-mediated knockdown of *TrMYB1* in *Tricyrtis* sp. ‘Shinonome’

**Fig. 4:**
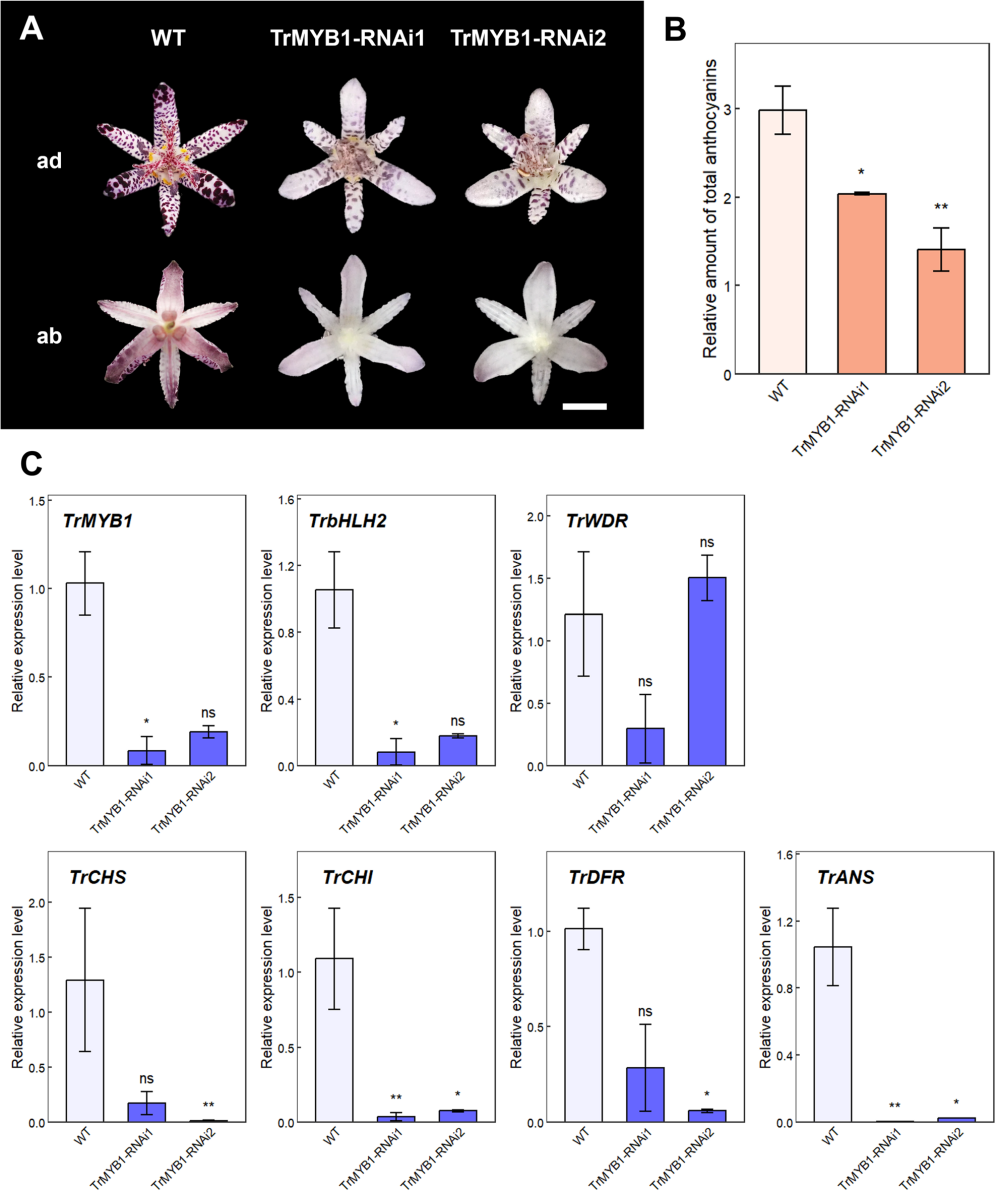
Effects of RNAi-mediated knockdown of *TrMYB1* in transgenic plants (**A**) Phenotypes of flowers of WT and transgenic plants. Bar = 1 cm. ab, abaxial side; ad, adaxial side. (**B**) Relative amounts of total anthocyanins in outer tepals of WT and transgenic plants. Data are shown as means ± SE (n = 3). * and ** indicate significant differences compared with WT at *p* < 0.05 and *p* < 0.01, respectively, by Dunnett’s test following one-way ANOVA. (**C**) Relative expression levels of anthocyanin biosynthesis-related genes in outer tepals of WT and transgenic plants by real-time RT-PCR. Data are shown as means ± SE (n = 3). * and ** indicate significant differences compared with WT at *p* < 0.05 and *p* < 0.01, respectively, by Dunnett’s test following one-way ANOVA. ns, not significant.

To further examine the function of *TrMYB1*, RNAi-mediated knockdown construct was introduced into *Tricyrtis* sp. ‘Shinonome’ (Fig. 2). Two independent transgenic lines (TrMYB1-RNAi1 and -RNAi2) were obtained and both produced flowers (Fig. 4A). In S5 flowers of both transgenic lines, background coloration on both the adaxial and abaxial sides of the tepals almost completely disappeared (Fig. 4A). By contrast, reddish-purple spots were still detectable, although their pigmentation intensity was markedly reduced compared with that in WT flowers (Fig. 4A). For both transgenic lines, the relative amounts of total anthocyanins in outer tepals of S5 flowers significantly decreased compared with WT (Fig. 4B). Real-time RT-PCR analysis using outer tepals of S5 flowers showed that the expression levels of *TrMYB1* in TrMYB1-RNAi1 and -RNAi2 were reduced to approximately 10% and 20% of that in WT, respectively (Fig. 4C). The expression levels of enzyme genes (*TrCHS*, *TrCHI*, *TrDFR*, and *TrANS*) and *TrbHLH2* were also substantially reduced in TrMYB1-RNAi1 and -RNAi2 (Fig. 4C). In particular, the expression levels of *TrCHI* and *TrANS* showed significant decreases in both transgenic lines (Fig. 4C). On the other hand, no significant changes in the *TrWDR* expression levels were observed in both transgenic lines (Fig. 4C).

## Discussion

To elucidate the molecular mechanism of flower color pattern formation in tepals of *Tricyrtis* sp., we previously performed comprehensive isolation and expression analyses of the anthocyanin biosynthesis-related genes^1,22^. In the present study, as a next step, we investigated the effect of light on anthocyanin biosynthesis in tepals and performed functional analysis of the R2R3-MYB gene *TrMYB1*, which may be involved in the regulation of anthocyanin biosynthesis in tepals.

Shading treatment of flower buds or inflorescences has been reported to reduce anthocyanin accumulation in tepals of several plant species, such as *G*. *hybrida*^9^, *C*. *morifolium*^11^, and *L*. *regale*^13^. Interestingly, background coloration in the tepals of *Tricyrtis* sp. was also largely reduced under shading conditions, whereas spot formation was scarcely affected (Fig. 1A). This differential response suggests that background coloration and spot formation in tepals are regulated by distinct mechanisms, in which background coloration is light dependent, whereas spot formation is largely light independent. Furthermore, the expression level of *TrMYB1* decreased under shading conditions (Fig. 1C), indicating that *TrMYB1* may be positively regulated by light. Consistent with this observation, several putative light-responsive cis-elements were identified in the *TrMYB1* promoter (Supplementary Fig. S3), suggesting that its transcription may be associated with light-responsive regulatory mechanisms. Together, these findings suggest that *TrMYB1* plays an important role in light-induced anthocyanin biosynthesis in tepals of *Tricyrtis* sp. Light-induced R2R3-MYB gene expression and anthocyanin biosynthesis have also been reported in other plant species, including *P*. *hybrida*^14,15^, *L*. *regale*^13^, and *C*. *morifolium*^16^, suggesting that similar regulatory mechanisms may be conserved across a wide range of higher plants.

In *Arabidopsis thaliana*, the anthocyanin biosynthesis–related R2R3-MYB transcription factors PAP1 and MYB12 are directly regulated by the light-responsive bZIP transcription factor HY5^23,24^. HY5 directly interacts with the ubiquitin ligase COP1 and is degraded in the nucleus under dark conditions. In contrast, COP1 is inactivated upon light exposure, preventing HY5 degradation and resulting in its stabilization^25^. COP1 also directly regulates the stability of the R2R3-MYB transcription factors PAP1 and PAP2^26^. Together, these light-dependent mechanisms regulate both the expression and protein stability of R2R3-MYB transcription factors, ultimately modulating anthocyanin biosynthesis. Similar regulatory mechanisms of light-induced anthocyanin biosynthesis mediated by COP1 and HY5 have been reported in other plant species, such as *M*. *domestica* and *S*. *melongena*^12^. Further studies are needed to clarify the molecular mechanism of light-induced *TrMYB1* expression in *Tricyrtis* sp.

In our previous studies, heterologous expression of *TrMYB1* increased anthocyanin accumulation in transgenic *Pelargonium crispum* and *Kalanchoe blossfeldiana*^27,28^, suggesting that *TrMYB1* functions as a conserved regulator of anthocyanin biosynthesis across species. To further clarify its role in its native genetic background, we generated transgenic *Tricyrtis* plants overexpressing *TrMYB1*. Overexpression of *TrMYB1* resulted in a significant increase in anthocyanin accumulation in the leaves (Fig. 3A, C). The expression levels of several anthocyanin biosynthetic enzyme genes (*TrCHS*, *TrCHI*, *TrDFR*, and *TrANS*) were also increased in the leaves of these transgenic plants (Fig. 3E), supporting the involvement of TrMYB1 in regulating anthocyanin biosynthesis. This result is consistent with our previous finding that TrMYB1 is associated with regulation of late biosynthetic genes^1^. The expression level of *TrbHLH2* was also increased in the leaves of these transgenic plants, suggesting a possible regulatory relationship between TrMYB1 and *TrbHLH2*, either directly or indirectly through regulatory feedback mechanisms. Similarly, overexpression of R2R3-MYB genes has been shown to activate the expression of bHLH genes in various plant species^29–32^. Although the coordinated expression patterns strongly suggest the involvement of TrMYB1 in anthocyanin biosynthesis, direct binding between TrMYB1 and the promoters of anthocyanin biosynthesis-related genes has not yet been demonstrated. Further studies will be necessary to determine whether TrMYB1 directly regulates these genes. In addition, overexpression of *TrMYB1* resulted in enhanced pigmentation in most floral organs (Fig. 3B). However, the absence of ectopic pigmentation in the basal region of the tepals in *TrMYB1*-overexpressing lines suggests that additional regulatory factors, such as bHLH and WDR transcription factors, may be required for anthocyanin accumulation in this region. By contrast, enhanced pigmentation was observed in regions that are already pigmented in the tepals of WT plants, implying that *TrMYB1* alone may be sufficient to enhance anthocyanin biosynthesis in such regions.

In transgenic *Tricyrtis* sp. plants with RNAi-mediated knockdown of *TrMYB1*, both anthocyanin accumulation and the expression levels of anthocyanin biosynthetic enzyme genes, as well as *TrbHLH2*, decreased in outer tepals (Fig. 4B, C). Moreover, the flowers of transgenic plants showed a pronounced reduction of background coloration on both the adaxial and abaxial sides of the tepals, whereas reddish-purple spots were still detectable (Fig. 4A). These results indicate that background coloration and spot formation may be regulated by different mechanisms, and that *TrMYB1* may play a central role in the formation of tepal background coloration through regulation of anthocyanin biosynthesis-related genes in *Tricyrtis* sp. The involvement of multiple R2R3-MYB transcription factors in the regulation of flower color pattern formation has been reported in several plant species. In *P*. *hybrida*, four R2R3-MYB genes have been identified as regulators of anthocyanin biosynthesis in tepals. Among them, *AN2* and *AN4* control pigmentation in the limb and tube of tepals, respectively^33,34^. In addition, *DPL* regulates veining in the petal tube, and *PHZ* is light inducible and associated with blushing flower buds^15^. In *Clarkia gracilis*, four R2R3-MYB genes involved in tepal pigmentation have also been identified^35^. Among them, *CgMYB1C* controls red spot formation, whereas *CgMYB12* regulates background coloration in the basal (cup) region. *CgMYB6* and *CgMYB11* are also suggested to contribute to background coloration. In the present study, spot pigmentation was clearly reduced in the TrMYB1-RNAi lines. It is possible that additional R2R3-MYB transcription factors are involved in the regulation of spot pigmentation and that their activities may have been partly affected by off-target effects of the RNAi construct. Taken together, these findings suggest that multiple R2R3-MYBs, including *TrMYB1*, may cooperatively regulate flower color pattern formation in *Tricyrtis* sp.

In the present study, we demonstrated that *TrMYB1* is expressed in response to light and regulates tepal background coloration by promoting anthocyanin biosynthesis. The results obtained here are expected to contribute significantly to studies on flower color pattern formation in ornamental plants. However, the molecular mechanism of tepal spot formation in *Tricyrtis* remains unclear. In particular, the mechanism underlying the formation of randomly distributed spots of various sizes has not yet been reported and represents a highly intriguing biological phenomenon. Therefore, elucidating the molecular basis of spot formation in *Tricyrtis* sp. may provide an important model system for understanding the general principles of flower color pattern formation, and further advances in this area are strongly anticipated.

## Materials and methods

### Plant materials and callus cultures

Potted plants of *Tricyrtis* sp. ‘Shinonome’ were obtained from commercial sources and cultivated in a greenhouse without heating at Niigata University. Embryogenic callus cultures used for transformation were induced and maintained as described by Nakano et al.^36^. No wild plant materials were collected for this study, and therefore no specific permits or licenses were required. The study did not involve endangered or protected species, and all experiments were conducted in accordance with relevant institutional guidelines.

### Shading treatment of flower buds

Shading treatment was carried out by covering shoot apices with aluminum foil from the time of bract emergence until anthesis. Outer tepals were collected from samples with and without shading treatment at five different developmental stages: S1, flower buds of 6–10 mm in length; S2, flower buds of 11–15 mm in length; S3, flower buds of 16–20 mm in length; S4, flower buds just before anthesis (over 21 mm in length); and S5, flowers just after anthesis^1^.

### RNA isolation and cDNA synthesis

Total RNA was extracted using RNeasy Mini Kit (QIAGEN, Hilden, Germany) and treated with RNase-Free DNase Set (QIAGEN, Hilden, Germany) according to the manufacturer’s instructions. cDNA was synthesized using ReverTra Ace qPCR RT Master Mix (Toyobo Co., Ltd., Osaka, Japan) according to the manufacturer’s instructions.

### RT-PCR and real-time RT-PCR

Reverse transcription-polymerase chain reaction (RT-PCR) was performed using EmeraldAmp MAX PCR Master Mix (Takara Bio Inc., Shiga, Japan) on the T100 thermal cycler (Bio-Rad, Hercules, CA, USA) according to Otani et al.^1^.

Real-time RT-PCR was performed using KOD SYBR qPCR Mix (Toyobo Co., Ltd., Osaka, Japan) on the MyGo real-time PCR system (IT-IS Life Science Ltd., Dublin, Ireland). The specificity of the amplification products was confirmed by melting curve analysis. Real-time RT-PCR was performed in biological triplicate for each sample. Relative amounts of transcripts were normalized to the actin gene of *Tricyrtis* sp. (*TrAct2*; AB196260) and calculated using the 2^−ΔΔCt^ method^37^.

Primer sets used for these analyses are listed in Supplementary Table S1 online.

### Measurement of total anthocyanins

Total anthocyanins were extracted from outer tepals of flowers at different developmental stages and from leaves using the methanol-HCl method according to Rabino and Mancinelli^38^. Absorbance of the extracts was measured at 530 and 657 nm using a spectrophotometer (Eppendorf BioSpectrometer basic; Eppendorf, Hamburg, Germany). Total anthocyanin contents were calculated with the following formula: (A_530_—0.25 × A_657_) × M^−1^ [M: weight (g) of the plant material used for extraction].

### Promoter isolation and cis-element analysis of *TrMYB1*

The upstream promoter region of *TrMYB1* was isolated by TAIL-PCR^39^. Cis-regulatory elements in the promoter sequence were identified using the PlantCARE database^40^. Putative light-responsive cis-elements were identified based on the database annotations.

### Vector construction and *Agrobacterium*-mediated transformation

*Agrobacterium tumefaciens* strains EHA101/pIG-*TrMYB1* and EHA101/p*TrMYB1*-RNAi were used to produce transgenic plants for *TrMYB1* overexpression and RNAi-mediated knockdown, respectively. The T-DNA region of pIG-*TrMYB1* contained *TrMYB1* driven by the CaMV 35S promoter, neomycin phosphotransferase II (*NPTII*) driven by the Nopaline Synthase (*NOS*) promoter, and hygromycin phosphotransferase (*HPT*) driven by the CaMV35S promoter (Fig. 2). The p*TrMYB1*-RNAi vector was constructed based on the pANDA35HK vector^41,42^. The T-DNA region of p*TrMYB1*-RNAi contained two inverted repeats of a 22-bp *TrMYB1* trigger sequence fragment driven by the CaMV35S promoter, *NPTII* driven by the *NOS* promoter, and *HPT* driven by the CaMV35S promoter (Fig. 2). All primers used for vector construction are listed in Supplementary Table S1 online.

Transformation of *Tricyrtis* sp. ‘Shinonome’ was performed as previously described by Adachi et al.^43^. To confirm the transgenic nature of regenerated plantlets, PCR analyses were performed using primer sets specific to the T-DNA region (Supplementary Table S1 online). Transgenic plantlets were acclimatized and cultivated in a growth room at 25 °C under continuous light-emitting diode (LED) lighting (ca. 170 μmol m^–2^ s^–1^). Morphological, biochemical, and molecular characterizations of transgenic plants were performed during the flowering season.

## Supplementary Information

Below is the link to the electronic supplementary material.

Supplementary Material 1

## Data Availability

The datasets generated during and/or analysed during the current study are available from the corresponding author on reasonable request.
