# Supplementary material for: Identification of an R2R3-MYB gene regulating tepal background coloration in Tricyrtis sp
Source: Sci Rep. 2026 Mar 28;16:10743. doi: 10.1038/s41598-026-46254-x (PMC13039122; doi:10.1038/s41598-026-46254-x)
Supplement: Supplementary file 1 — Supplementary Material 1 [file 41598_2026_46254_MOESM1_ESM.pdf]

Supplementary information

Title of the paper:

Identification of an R2R3-MYB gene regulating tepal background coloration in *Tricyrtis* sp.

Names of the authors:

Yuta Shinoku, Ichiro Kazama, Yusuke Kanemaki, Mai Shibuya, Kakeru Inagawa, Juria Ono, Masaru Nakano, Masahiro Otani

Corresponding author:

Masahiro Otani, Ph.D.

Faculty of Agriculture, Niigata University,

2-8050 Ikarashi, Nishi-ku, Niigata 950-2181, Japan.

E-mail: otani@agr.niigata-u.ac.jp

TEL, FAX: +81-25-262-6680

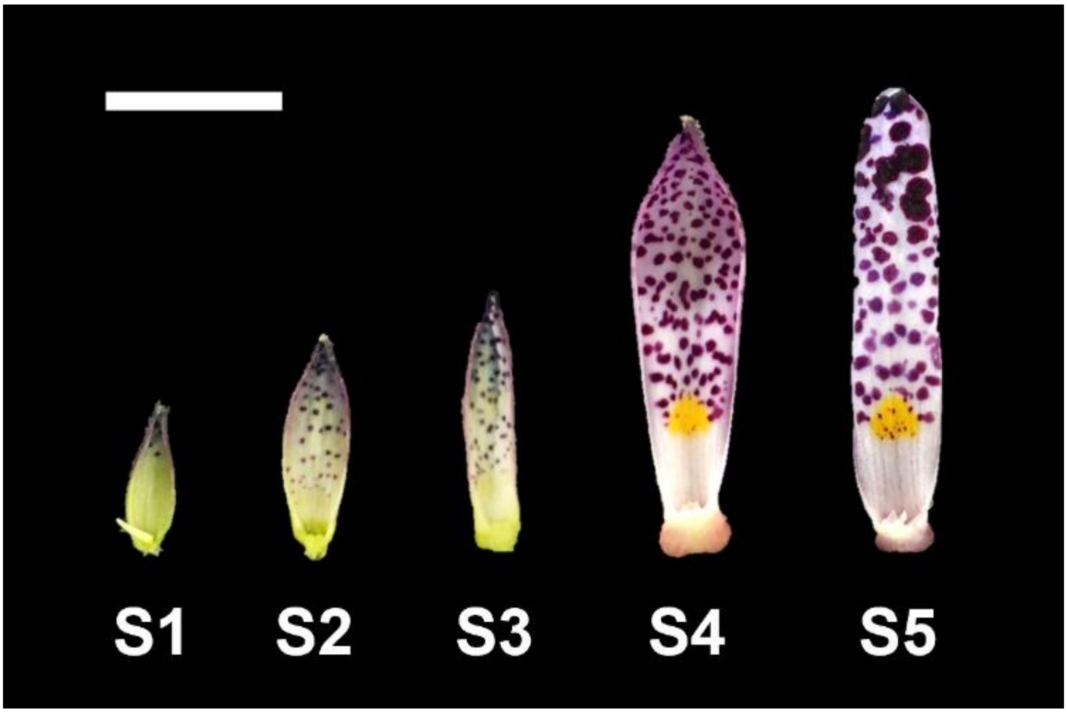

Figure S1. Flower pigmentation phenotypes on the adaxial side of the outer tepals at each developmental stage. Bar = 1 cm.

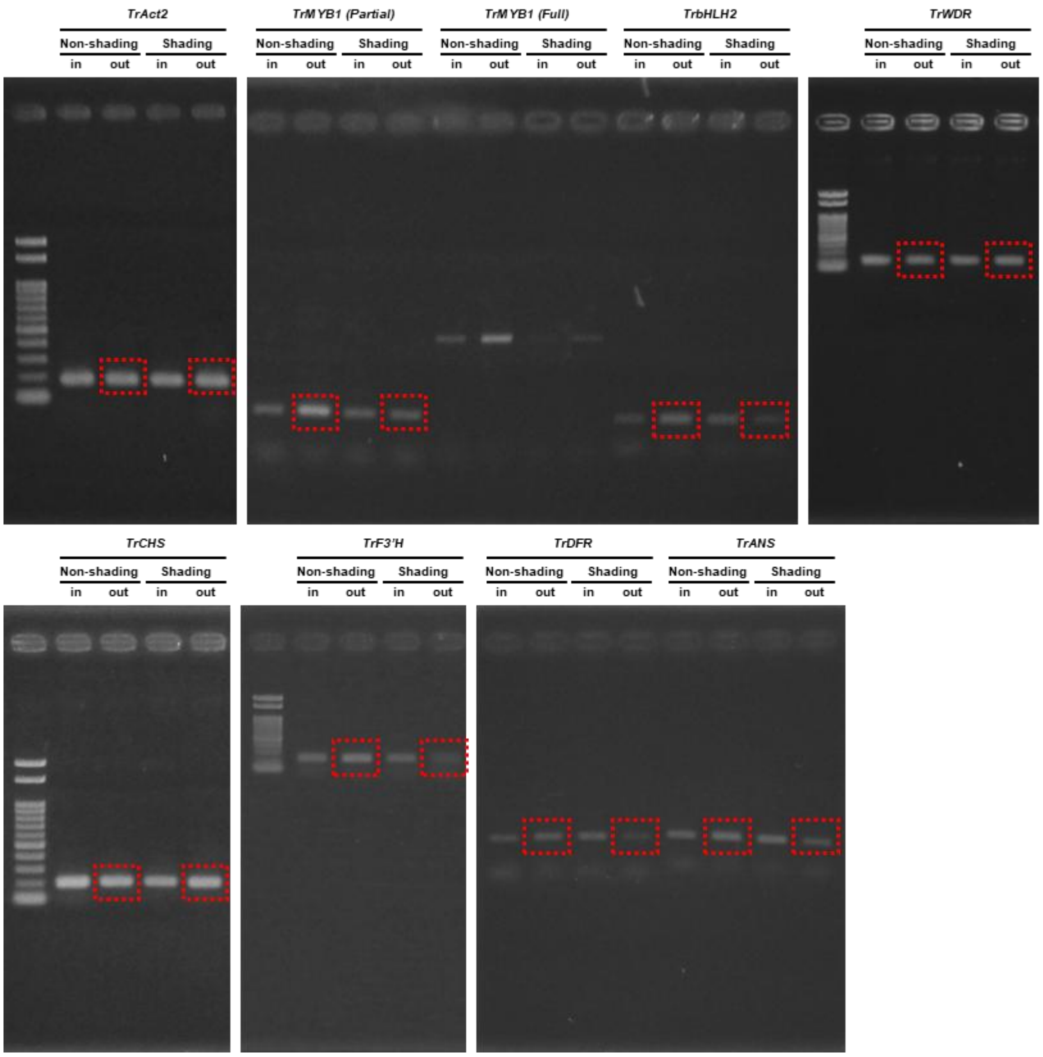

Figure S2. Full-length gel images corresponding to Fig. 1C. Cropped regions are indicated by red boxes. in, inner tepals; out, outer tepals.

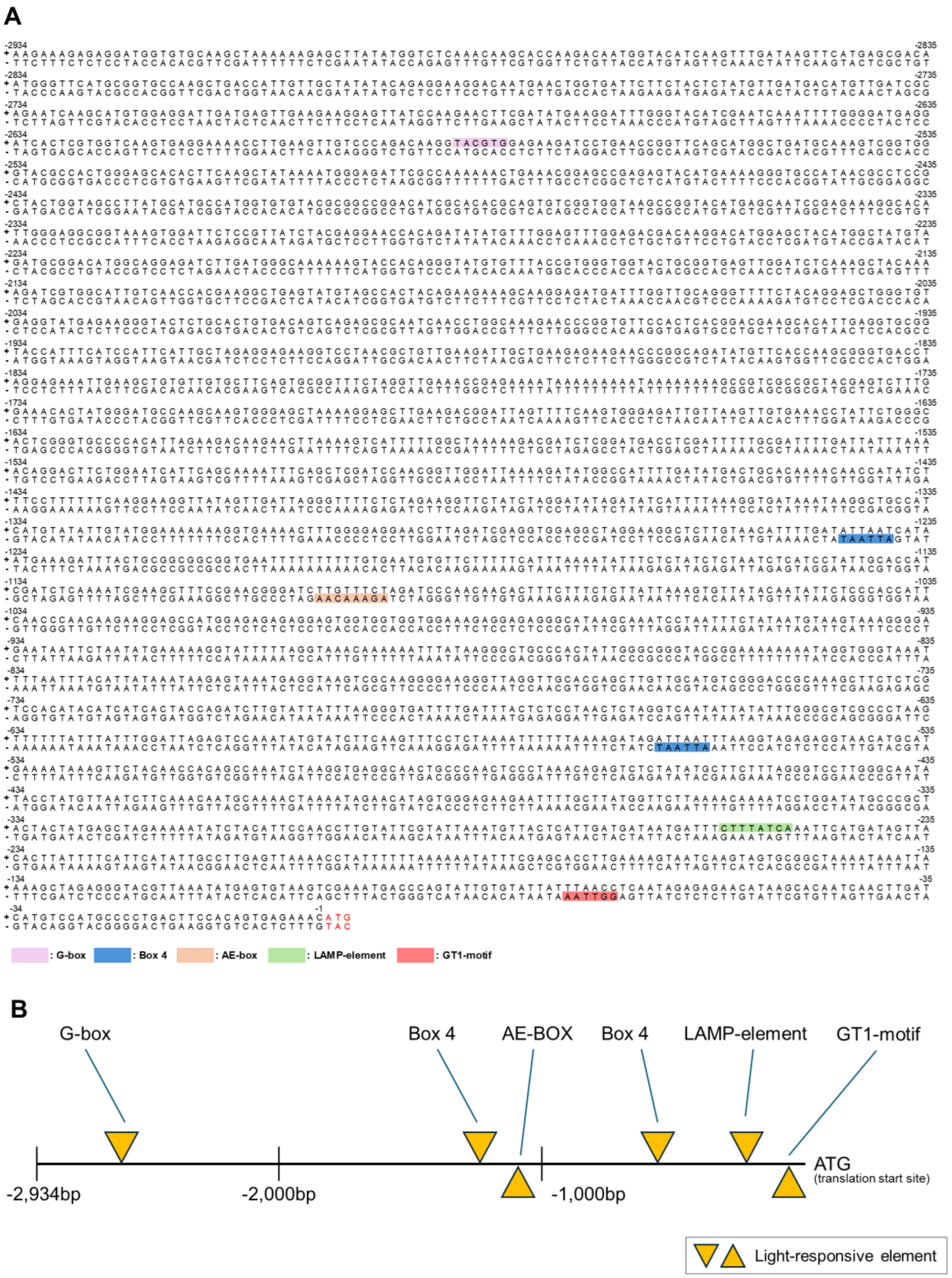

Figure S3. Identification of putative light-responsive cis-elements in the *TrMYB1* promoter. (A) Predicted light-responsive cis-elements in the upstream promoter region of *TrMYB1* (−2,934 bp relative to the translation start site), identified using the PlantCARE database. Light-responsive motifs are highlighted. (B) Schematic diagram of the distribution of light-responsive cis-elements in the *TrMYB1* promoter. Yellow triangles indicate the positions of light-responsive cis-elements, including G-box, LAMP-element, AE-box, Box 4, and GT1 motif**.**

Table S1. Primer sets used in the present study

Primer name	Sequence (5’->3’)	Note
TrMYB1_RT-PCR_Fw<br>TrMYB1_RT-PCR_Rv	CTACTCCGGAGATGCGTTGAGAACC<br>CAGTCCTACCGGGAATTCGACCGGC	For RT-PCR
TrMYB1_qPCR_Fw<br>TrMYB1_qPCR_Rv	TTGAGCAGCACCAAGAAGATGC<br>TGCTCGAAGTCGTTTGGTTGC	For qRT-PCR
TrbHLH2_RT-PCR_Fw<br>TrbHLH2_RT-PCR_Rv	GCAGATGGAGAACAATCAGAGGTC<br>CACATTGGTACTGAAAGCCTCTACTGC	For RT-PCR and qRT-PCR
TrWDR_RT-PCR_Fw<br>TrWDR_RT-PCR_Rv	AGCAAGGTGCAACTCTTGAGG<br>CACGCTGGAAATCCACACTC	For RT-PCR and qRT-PCR
TrCHS_RT-PCR_Fw<br>TrCHS_RT-PCR_Rv	TGGAGGTTAAGTTGGCGTTG<br>CGCTTCCTCATCTCATCCAG	For RT-PCR and qRT-PCR
TrCHI_RT-PCR_Fw<br>TrCHI_RT-PCR_Rv	CCCTCCTGGTGCCTCCATCCTCTTC<br>CGCTAATGGCAATTGGCTCCTTAGC	For RT-PCR and qRT-PCR
TrF3’H_RT-PCR_Fw<br>TrF3’H_RT-PCR_Rv	GCGGCGGAGGAGTTTAAGGAGATGG<br>GGCCCATTCTACTGTGCTCGAGG	For RT-PCR
TrDFR_RT-PCR_Fw<br>TrDFR_RT-PCR_Rv	CAATGCCACCCAGTATGATAACC<br>CATGAAAAGAACACATATATCTCCC	For RT-PCR
TrDFR_qPCR_Fw<br>TrDFR_qPCR_Rv	CATGTCGCCACTCCGATGGA<br>ACCGTCCCTGCTTTCTTGCA	For qRT-PCR
TrANS_RT-PCR_Fw<br>TrANS_RT-PCR_Rv	GACAATTGTGTTGAAGCCGTTG<br>GGCTCCAACCGAAGATTTTATTG	For RT-PCR and qRT-PCR
TrAct2_RT-PCR_Fw<br>TrAct2_RT-PCR_Rv	TGCCATGTATGTTGCCATTCAG<br>AGGGAGTCGGTCAGGTCTCTG	For RT-PCR and qRT-PCR
TrMYB1_cDNA_Fw<br>TrMYB1_cDNA_Rv	GCCCCTGACTTCCACAGTGAG<br>CGGCAGCAATCCACGCTC	For cloning into pMD20
TrMYB1_RNAi_Fw<br>TrMYB1_RNAi_Rv	GATCATTTTGCAAGAAGAACTGGCAC<br>AATTGTGCCAGTTCTTCTTGCAAAAT	For construction of RNAi-mediated knockdown vector
35Spro150_Fw<br>TrMYB1_Rv	TCCAACCACGTCTTCAAAGC<br>CGGTTCAGGTTCAGTGAGGTGGA	For confirmation of transformation with the overexpression construct
HPT290_Fw<br>HPT290_Rv	GTGCTTTCAGCTTCGATGTAGG<br>GCTCGTCTGGCTAAGATCGG	For confirmation of transformation with the RNAi construct
